# A taguchi neural network–based optimization of a dual-port, dual-band MIMO antenna encompassing the 28/34 GHz millimeter wave regime

**DOI:** 10.1038/s41598-025-90103-2

**Published:** 2025-02-19

**Authors:** Ajay Kumar Dwivedi, Vivek Singh, Yazeed Alzahrani, R Krishna Chaitanya, Suyash Kumar Singh, Subhav Singh, Komal Parashar, Manoj Tolani

**Affiliations:** 1 Department of Electronics and Communication Engineering, Nagarjuna College of Engineering and Technology, Bengaluru, India; 2 Department of Computer Engineering and Information, College of Engineering, Prince Sattam Bin Abdulaziz University-Wadi Addwasir, Wadi Addwasir, Saudi Arabia; 3 Department of Electronics and Communication Engineering, Sagi Ramakrishnam Raju Engineering College, Chinnamiram, Bhimavaram, Andhra Pradesh India; 4 Department of Electronics & Communication, Indian Institute of Information Technology Allahabad, Prayagraj, Uttar Pradesh India; 5 Chitkara Centre for Research and Development, Chitkara University, Himachal Pradesh, 174103 India; 6 Centre of Research Impact and Outcome, Chitkara University, Rajpura, 140417 Punjab India; 7 Department of Information and Communication Technology, Manipal Institute of Technology, Manipal Academy of Higher Education, Manipal, Karnataka India

**Keywords:** Electrical and electronic engineering, Electronic and spintronic devices

## Abstract

This study presents a novel printed antenna design that operates at the millimeter-wave frequencies of 28 and 34 GHz, which are crucial for the current and upcoming mobile communication generations. The radiating component in the antenna is a slot-etched rectangular ring that is fed through a stepped impedance microstrip line feed. Using advanced machine learning techniques, the design parameters of the suggested antenna have been fine-tuned to ensure optimal impedance matching at 28 GHz within the frequency range of 27.61–28.49 GHz. Additionally, the antenna also provides excellent impedance matching at 34.5 GHz within the frequency range of 33.61–34.27 GHz. Using the designated antenna, a Multiple Input Multiple Output (MIMO) system with two ports is constructed. The MIMO system’s performance is evaluated by analyzing channel capacity loss (CCL), diversity gain (DG), and envelope correlation coefficient (ECC), which showcases outstanding outcomes. The study further explores the optimization of a antenna’s structure using a Taguchi-based Neural Network (Taguchi NN) approach to predict the reflection coefficient (|S_11_|) across a frequency range of 27–35 GHz. By systematically varying the gap width (∆w) and shift (∆t), a dataset was generated and used to train the network. The optimal model configuration achieved a validation Mean Square Error (MSE) of 2.244 and an R² of 0.848 enabling reliable prediction of the reflection coefficient (|S_11_|) without extensive simulations. The findings further highlight the construction and experimental assessment of a single-element antenna and MIMO system, which exhibit excellent impedance matching across both lower and higher frequency bands. The antenna displays a maximum gain of 8.75 and 5.5 dBi at frequencies of 28 and 34 GHz, respectively. The recommended antenna exhibits excellent radiation efficiency across both lower and higher frequency bands, with rates of 98.46% and 99.17%, respectively. In addition, the experimental measurements of the coupling coefficients between the MIMO antenna systems indicate extremely low coupling values. This results in an efficient MIMO system that is well-suited for future millimeter-wave (mm-wave) applications.

## Introduction

The increasing need for fast wireless communication has resulted in the advancement of using the millimeter-wave (mm-wave) frequency spectrum, which ranges from 30 to 300 GHz. Researchers have shown substantial interest in communication across the 28 GHz and 34 GHz spectra among these bands. This is because these spectra provide vast bandwidth, quick data transfer, and low absorption rate^1^. The frequency bands of 28-GHz and 34-GHz provide higher data rates and enhanced network capacity, making them well-suited for 5G and future systems. Antennas operating in these frequency bands need meticulous design to address obstacles such as increased signal loss during transmission and greater coverage areas often seen in millimeter-wave communications^2^. In addition, they must satisfy other criteria such as being small in size, having higher amplification, and having enhanced radiation patterns, all while maintaining effective power transmission and reception.

MIMO antenna technologies have facilitated the advancement of communication systems. These systems utilize the coordinated operation of multiple antennas to improve capacity and facilitate fast data transmission. MIMO antenna systems offer numerous advantages, such as increased spatial diversity and improved reliability of wireless communication by reducing fading and interference effects^3,4^. MIMO antenna systems operating in the millimeter-wave frequency range are an essential technology in 5G networks.

A major obstacle in MIMO antenna systems is the coupling between antennas, which may result in interference among the closely positioned antenna parts. This interference can cause a decline in signal quality, a rise in disturbance, and a decrease in Channel capacity^5^. The isolation between parts in a MIMO system is a significant concern. Increasing the height is necessary in order to reduce the coupling between the parts of the MIMO system and hence improve the overall performance. Recent research has extensively investigated strategies to reduce coupling in MIMO antennas. Various methods have been developed to reduce the interference between neighboring antenna components. Out of these several ways, the band-pass filter (BPF) is used to specifically choose a particular frequency range^6^. Electromagnetic band-gap (EBG), stub, microstrip resonator, meander-line, neutralization line, shorting pin, and meta surfaces are used in antenna design^7–15^.To improve isolation between components of stacked patch antenna arrays in MIMO applications, a new design termed a compact stack EBG was created. Effective decrease of mutual coupling across high-frequency bands was proven by this strategy^7^. In order to achieve substantial coupling suppression in CPW-fed two-port UWB MIMO antennas, the research created a new E-plane H-shaped EBG structure to increase isolation^8^. To improve the performance of air-gap-based MIMO antennas for 5G applications, a dual-band EBG structure was used to handle mutual coupling^9^. The study investigated the use of stub loading to reduce mutual coupling in UWB/MIMO antennas. Stubs were deliberately positioned to interrupt the current flow between adjacent antenna components, resulting in significant enhancements in isolation^10^. For MIMO antenna systems, the mitigation of mutual coupling was suggested using a meta-surface antenna array decoupling (MAAD) approach. To lessen interference, this novel method successfully redirected electromagnetic radiation^11^. An electromagnetic bandgap (EBG) structure including a meander line was used to diminish mutual coupling in ultra-wideband (UWB) multiple-input multiple-output (MIMO) antennas, specifically in the E-plane. The method demonstrated efficacy in separating antenna components while maintaining the overall compactness of the system^12^. This study presented Wang-shaped neutralisation lines to mitigate mutual coupling in MIMO antennas. The technology improved isolation by neutralising interfering currents among antenna components while preserving compactness^13^. The use of shorting pins was examined for the improvement of isolation in MIMO antennas. This method efficiently lowered coupling currents and enhanced isolation in small designs by connecting the patch parts to the ground plane^14^. A CLL (Complementary-Letter-L) metamaterial superstrate was used over a patch antenna array to reduce mutual coupling. This novel approach attained substantial isolation enhancements for MIMO applications, especially in high-density setups^15^. Important to a wireless network is the arrangement of its antennas. Consequently, a simple approach to antenna parameter optimization and design is required. The time required to complete high-frequency simulations has grown significantly due to technical developments such as downsizing and an increase in antenna parameters such as frequency and complexity. When faced with such problems, using machine learning models is the norm. In recent years, machine learning (ML) and artificial neural networks (ANN) have become valuable methodologies for developing and fine-tuning microwave components. Their application to various microwave circuits and antenna systems, including modeling and optimization, has demonstrated notable improvements in performance and efficiency. Moreover, these techniques have contributed to shortening design cycles and reducing costs^16–24^. Quick estimations of antenna parametric analysis are provided by these simulations^25^.

The current study relies on a composite antenna configuration consisting of a slot-etched rectangular ring-printed antenna. This antenna configuration enables the functioning of the frequency bands of 28 GHz and 34 GHz. The antenna parameters are tuned to provide an ideal antenna design, taking into account factors such as impedance bandwidth, gain, and efficiency. An antenna system with two ports and multiple-input multiple-output (MIMO) technology is implemented, using an arrangement of antenna components that are in front of each other. An analysis is conducted to evaluate the performance of both the single antenna and the MIMO antenna system. This study is novel since it is one of the few research efforts using a Taguchi-based Neural Network (Taguchi NN) for optimizing antenna design. This method substantially decreases computing expenses and duration while maintaining high precision in forecasting essential antenna characteristics such as the reflection coefficient (∣S_11_∣). The structure of the paper is as follows. Section II introduces the structural composition of the proposed dual-band MIMO antenna design. Section III provides an explanation of the single-element design of the intended MIMO system and 2-port MIMO antenna configuration. Sector IV explores the examination and practical aspects of the fabrication and experimental activities. Ultimately, the findings of the study are succinctly outlined in the conclusion section.

## Antenna configurations

**Fig. 1:**
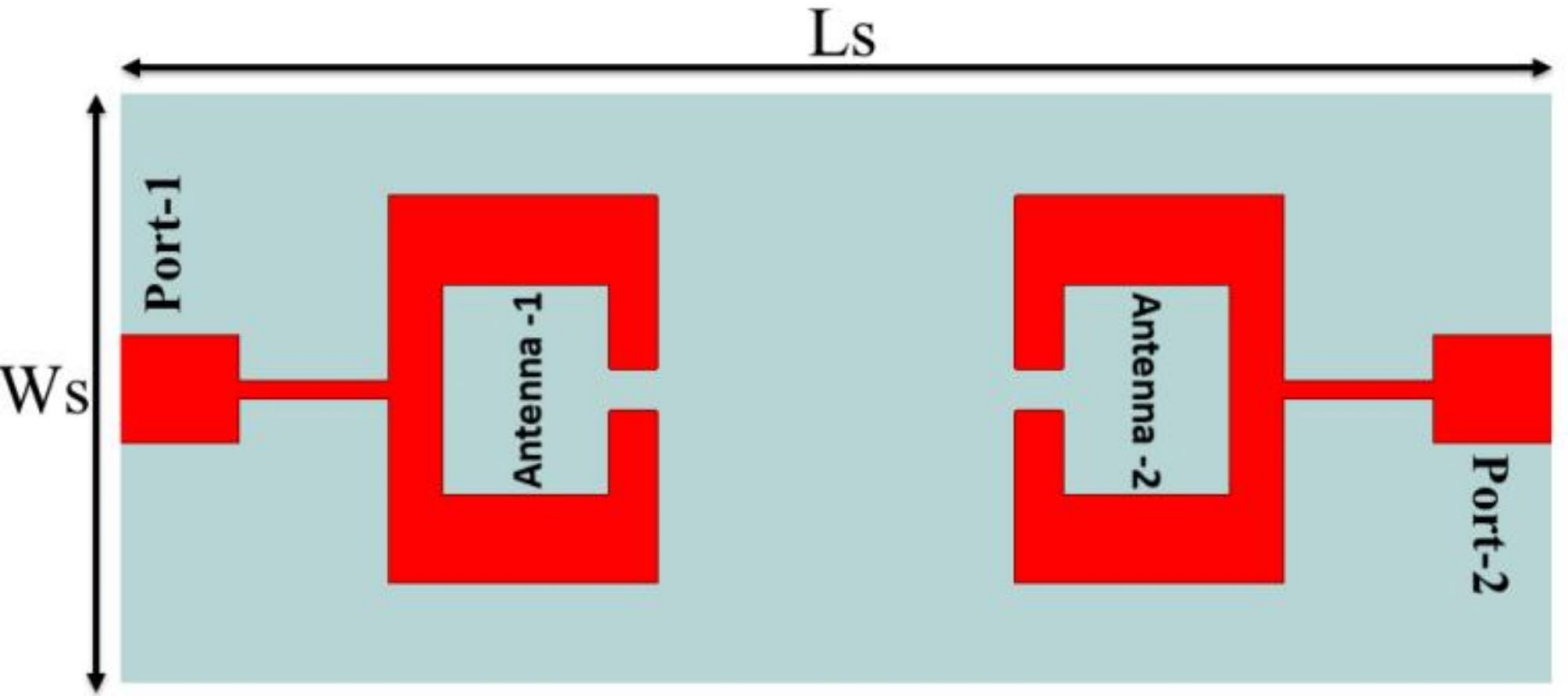
Proposed 2-Port MIMO Antenna.

**Fig. 2:**
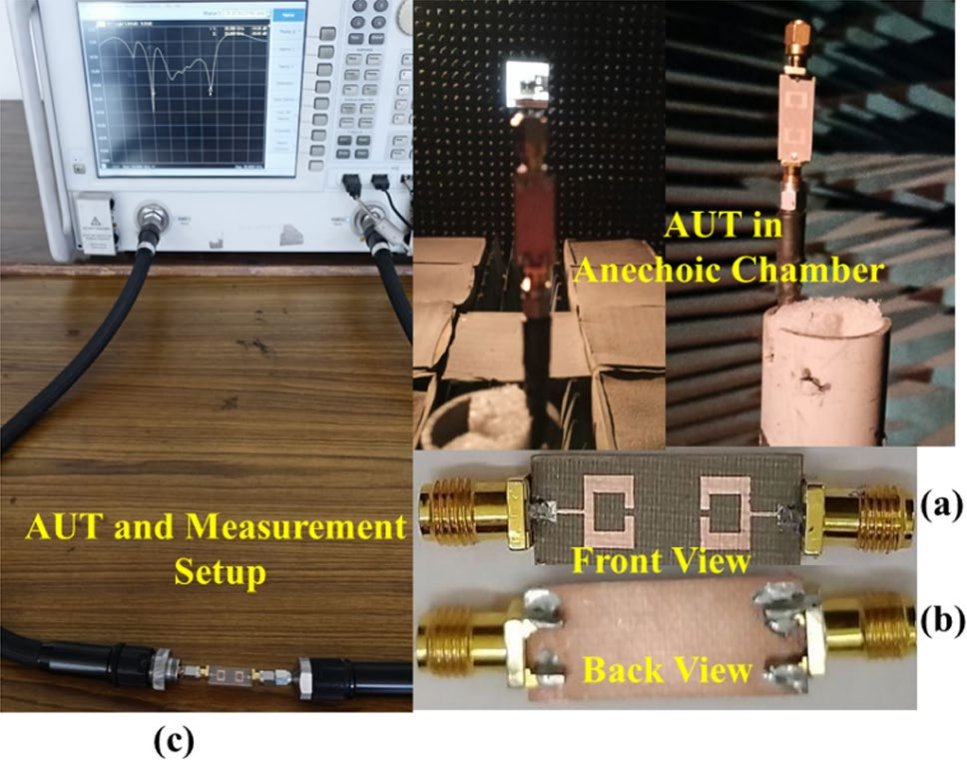
Fabricated model: (a) Front View; (b) Back View; and (c) With the Measurement Setup.

Figure 1 illustrates the structural layout, while Fig. 2(a, b, and c) showcases the constructed prototype of the proposed concept. The proposed model consists of a planar dual port MIMO antenna with a stepped feed line. The MIMO antenna is printed on a duroid 5880 substrate with a dielectric constant (εr) of 2.2, loss tangent (tanδ) of 0.0009, and thickness of 0.508 mm. The individual antenna element consists of an open-ended rectangular ring patch antenna, which is replicated in a front-to-front way to create the dual port MIMO antenna. The length (Ls) and Width (Ws) of the proposed MIMO antenna are 24 mm and 10 mm respectively.

## Analytical description of proposed design

### Single antenna design and evolution stages

**Fig. 3:**
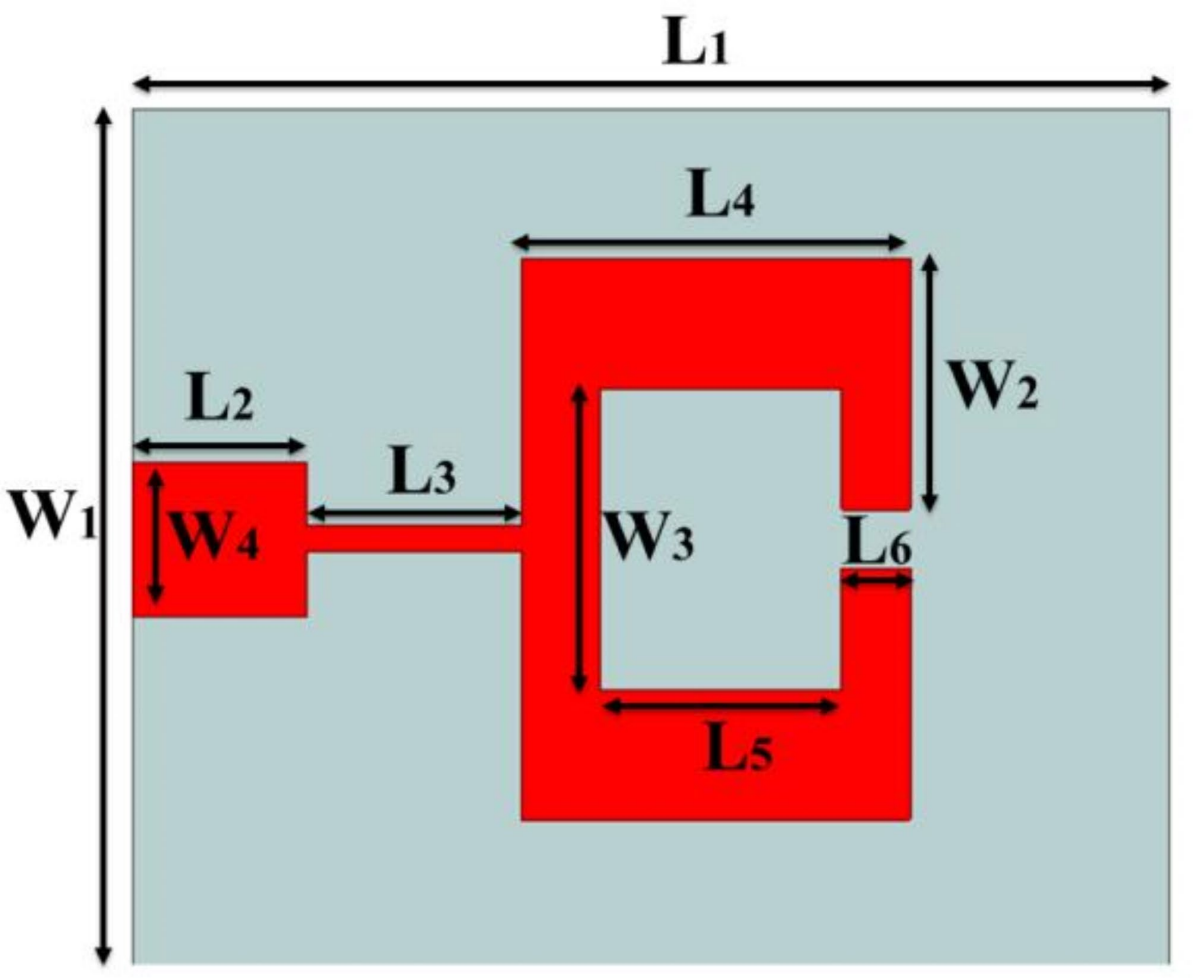
Single Antenna Element.

The structural composition of the single antenna element is presented in Fig. 3. The dimensional specification of the antenna is mentioned in Table 1.

**Table 1:** Dimensional specification of the single antenna element.

Parameters	Values (in mm)	Parameters	Values (in mm)
L1	12	W1	10
L2	2	W2	2.9
L3	2.5	W3	3.5
L4	4.5	W4	1.8
L5	2.8	Height of substrate (H)	0.508

**Fig. 4:**
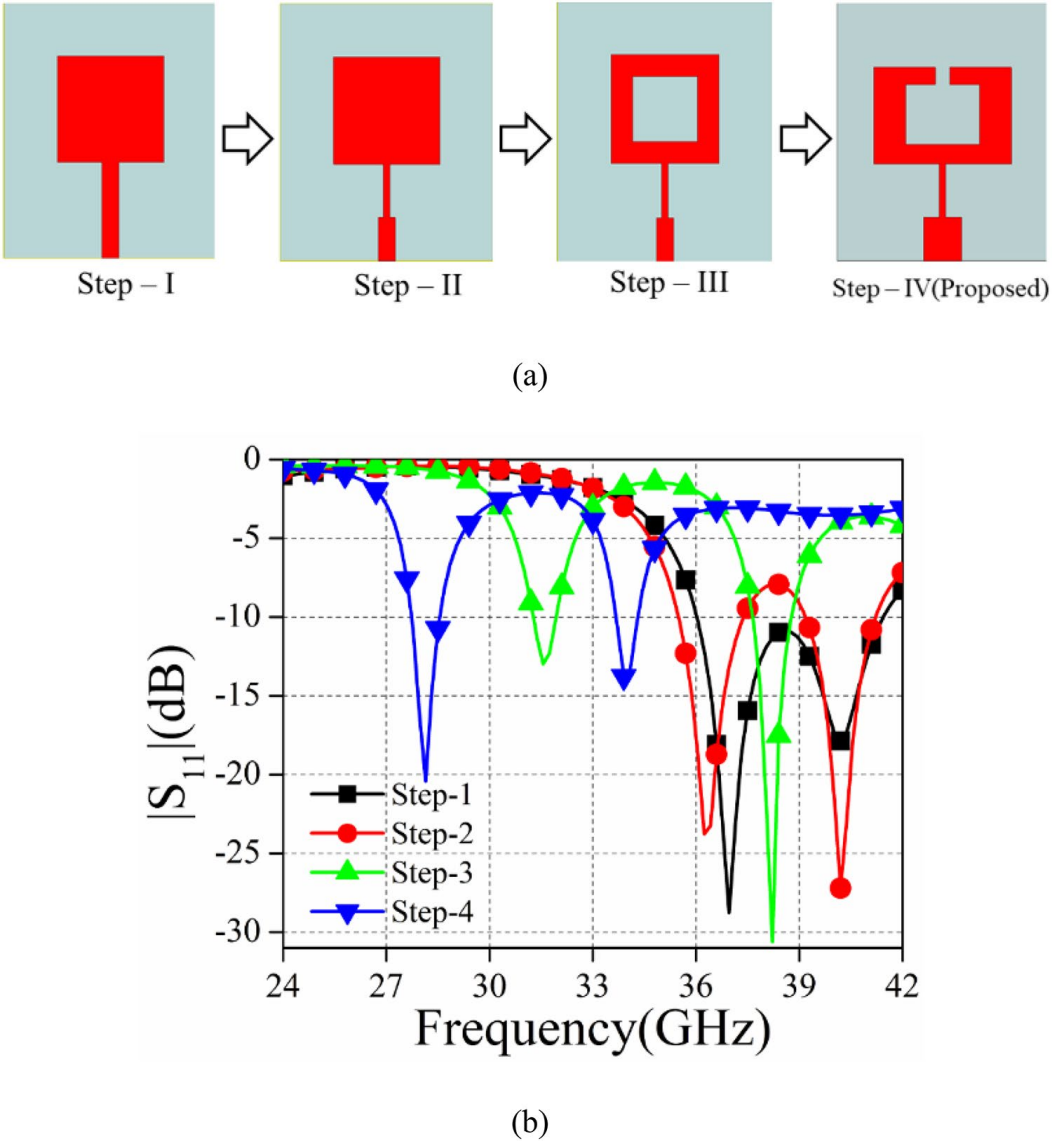
The evolution stages of the single antenna element (a) Step-wise structural layout, (b) |S_11_| plot of the different stages.

This section aims to analyze and investigate the different stages of evolution for the recommended single antenna component. The process of developing the proposed antenna involves four specific steps, namely step-I to step-IV. Throughout this process, a comparative investigation will be conducted, focusing on the |S_11_| (dB) values as shown in Fig. 4. Upon examining Fig. 4(b), it has been noted that all the stages of antenna evolution (Step-I to Step-IV) exhibit dual band characteristics except step-I which is wideband. Step I involve the utilization of a basic rectangular patch that is fed with a microstrip line, resulting in a wideband response. The second step involves the conversion of the simple microstrip feed line into a stepped impedance feed line, resulting in the manifestation of dual-band behavior. In order to obtain step-III, a rectangular slot has been etched out from the step-II layout, resulting in a shifted dual band in the lower frequency range. Ultimately, the parametric analysis has been conducted in the step-III configuration in order to achieve dual-band resonance at 28/34 GHz. For the purposes of this parametric analysis, modifications have been made to the width of the ring and a rectangular slot has been created by etching out a portion of the radiator.

## Machine learning algorithms

**Fig. 5:**
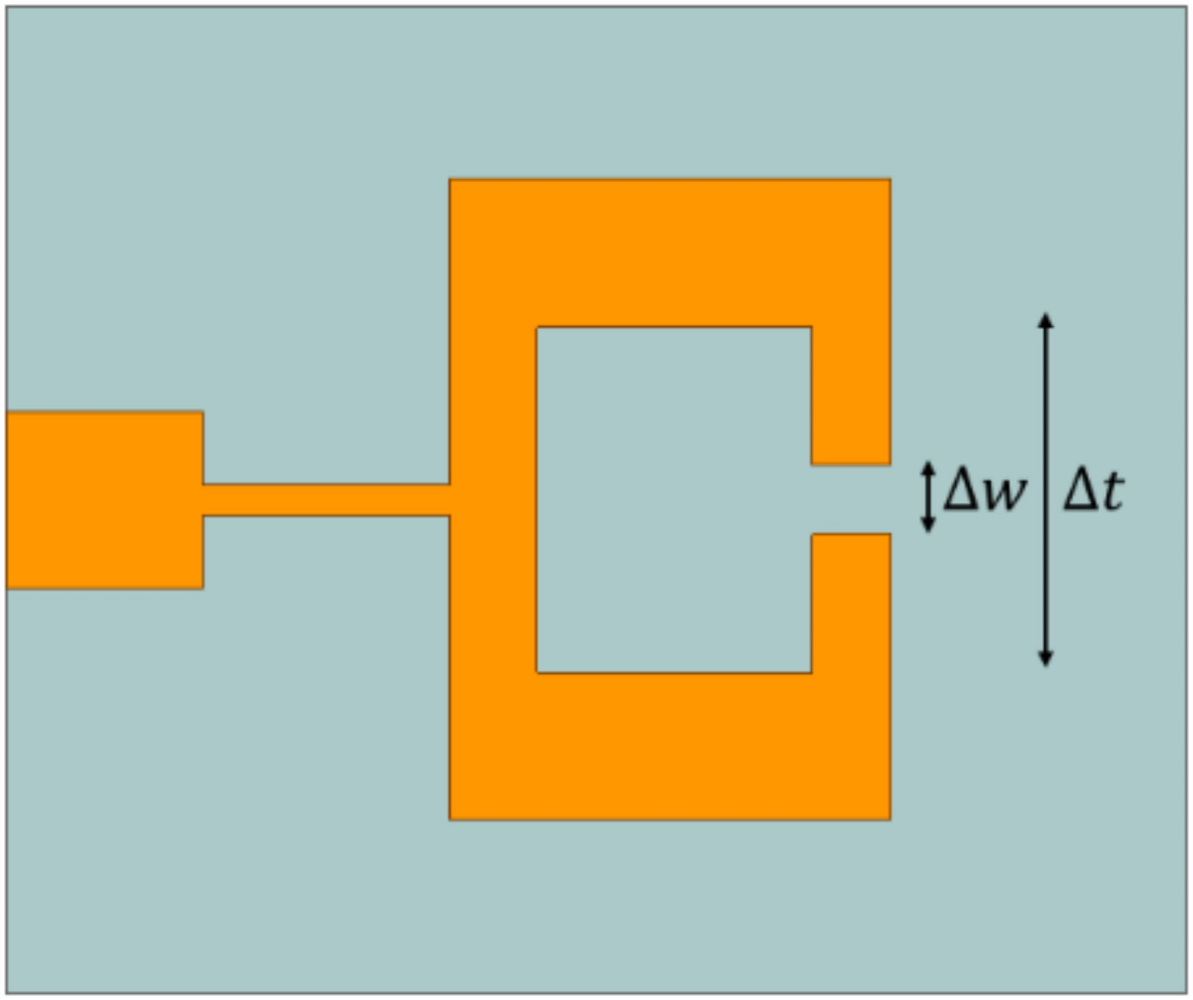
Unit cell for the extraction of the datasets.

A robust T-ANN model for predicting the reflection coefficient relies on a comprehensive dataset. In this work, such a dataset was obtained by conducting 8,000 full-wave electromagnetic simulations using the Ansys Electronics Suite. These simulations were specifically designed to generate accurate reflection coefficient ($\:{S}_{11})\:$data. To ensure proper wave propagation and confinement, the unit cell’s boundary conditions included copper on the top and bottom to represent the metallic structure. During the simulations, two key geometric parameters—the gap width (∆w) and its shift along (∆t)—were systematically varied due to their pronounced impact on the reflection coefficient ($\:{S}_{11})$ as shown in Fig. 5. The width ∆w was varied from 0.2 mm to 1.5 mm in increments of 0.1 mm, while the shift ∆t was adjusted from 3.2 mm to 5.95 mm in increments of 0.2 mm. These variations with dataset of 6220 values were conducted across a frequency range of 27 GHz to 35 GHz to form independent datasets. The corresponding reflection coefficient |S_11_| values, with a threshold of less than − 10 dB, were recorded to form the dependent dataset. This dataset was then used for further analysis and optimization to ensure optimal performance within the specified frequency band.

**Fig. 6:**
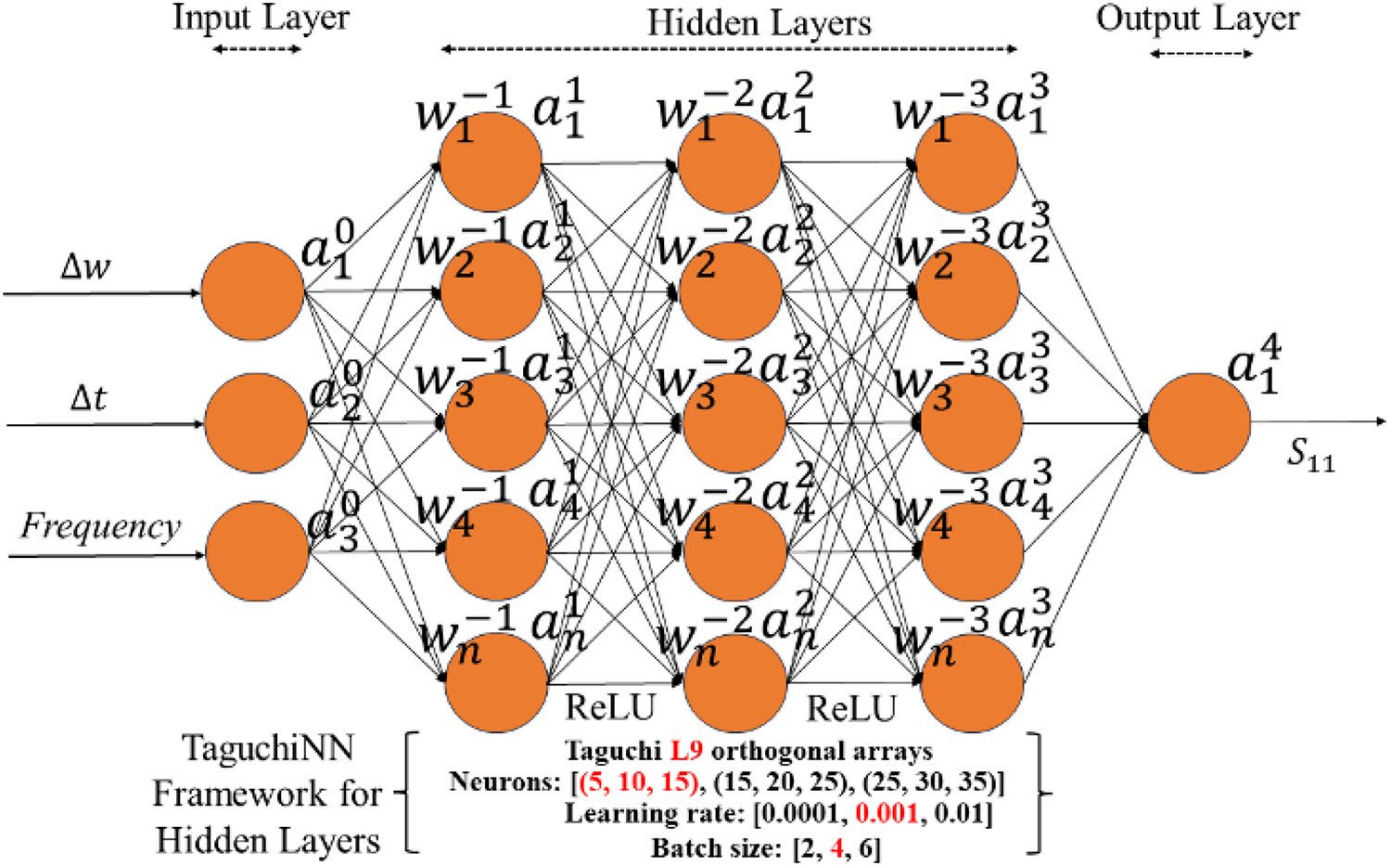
Taguchi neural network framework for the specified independent layers to predict the reflection coefficient.

In the illustrated framework as shown in Fig. 6, a Taguchi-based Neural Network (Taguchi NN) was employed. The Taguchi-based Artificial Neural Network (T-ANN) presented in this study builds upon the conventional ANN/DNN framework. By integrating the L9 orthogonal array, T-ANN systematically optimizes key hyperparameters—such as batch size, number of neurons, and learning rate—without resorting to the manual trial-and-error process commonly used in traditional methods. Unlike standard ANN, which requires tuning each parameter for every configuration and incurs a high computational cost, T-ANN narrows the search from $\:{L}^{n}\:$possible settings (where L = 3 levels per feature and *n* = 3 features in this work) to just nine. As a result, it rapidly identifies the best hyperparameter combination, leading to significant gains in both computational efficiency and overall performance. Further, the independent features, including the gap width (∆w), the gap shift ∆t, and the frequency f, were used as inputs to the network. The reflection coefficient (|S_11_|) was used as the dependent variable. The network’s hidden layers were optimized using the Taguchi L9 orthogonal array, with the with three level of neurons in each layer and three level of learning rate and batch size as shown in Table 2. The ReLU activation function was applied across the hidden layers, and the learning rate and batch size were systematically varied as part of the Taguchi experimental design to achieve optimal performance.

**Table 2:** L9 Taguchi Levels.

Learning rate	5,10,15	15,20,25	20,25,30
Batch size	10	20	30
Learning rate	0.001	0.01	0.1

**Fig. 7:**
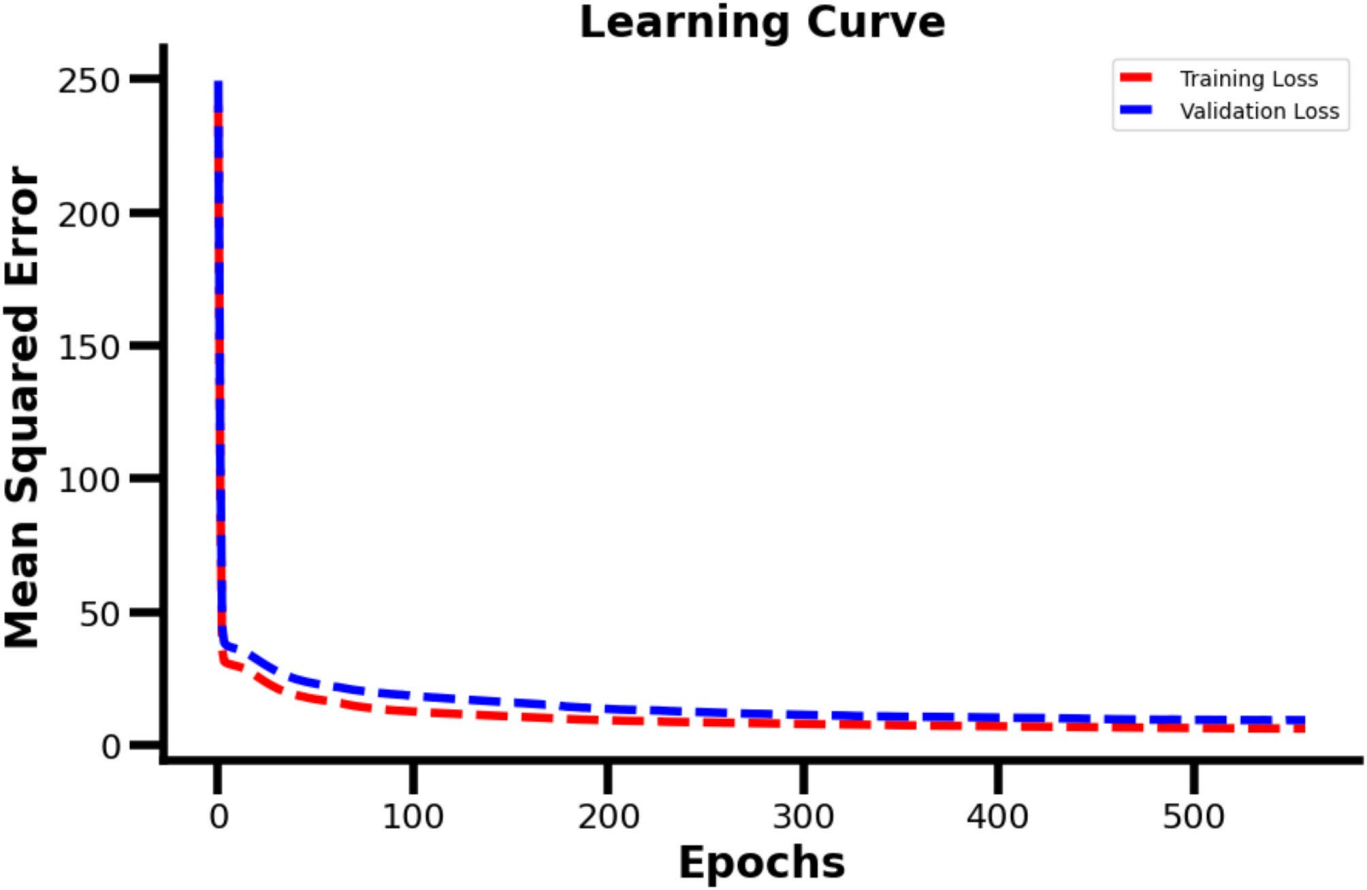
Mean squared error loss function with respect to number of epochs.

The performance of the model was evaluated using various configurations, and the optimal results were obtained with the following parameters: a neuron configuration of (15, 20, 25) across the hidden layers, a learning rate of 0.001, and a batch size of 20. Under these settings, the model achieved a validation Mean Squared Error (MSE) of 2.244 and converged with increasing number of epochs as shown in Fig. 7, indicating the average squared difference between the predicted and actual values.

**Fig. 8:**
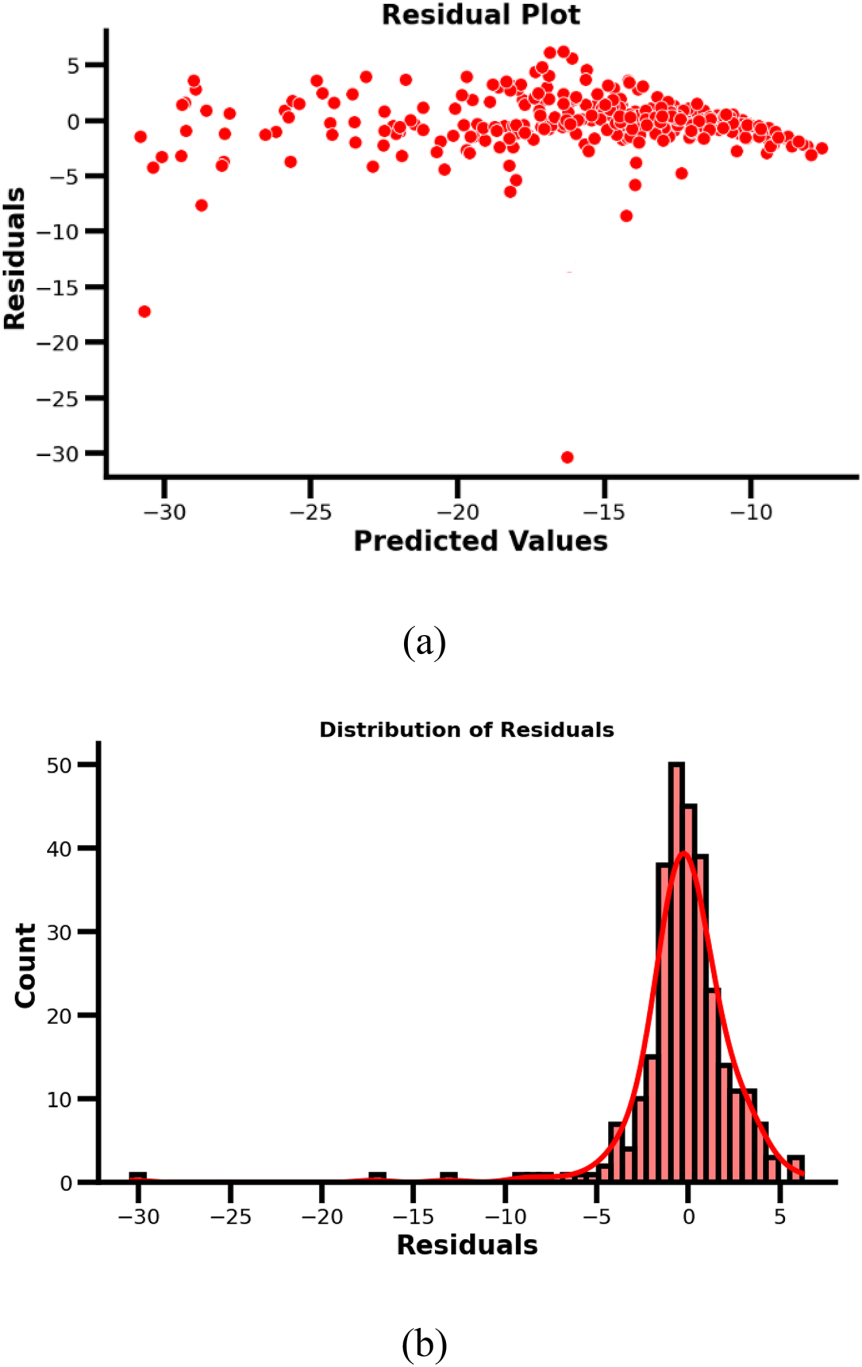
(a) Residual |S_11_| with respect to predicted |S_11_| (b) Normal distribution of residual count with respect to residuals.

Additionally, the model obtained a validation R^2^ score of 0.848, reflecting that approximately 84.8% of the variance in the dependent variable (|S_11_|) was explained by the model. These metrics suggest a good fit of the model to the validation data. The residuals plot as shown in Fig. 8(a), representing the differences between the predicted and observed values (|S_11 predicted_ - |S_11 observed_), are plotted on the y-axis, while the predicted values (|S_11 predicted_) are on the x-axis. Apart for a few outliers most of the residuals lies in the range ± 5dB which can be further exhibited distribution plot as shown in Fig. 8(b).

**Fig. 9:**
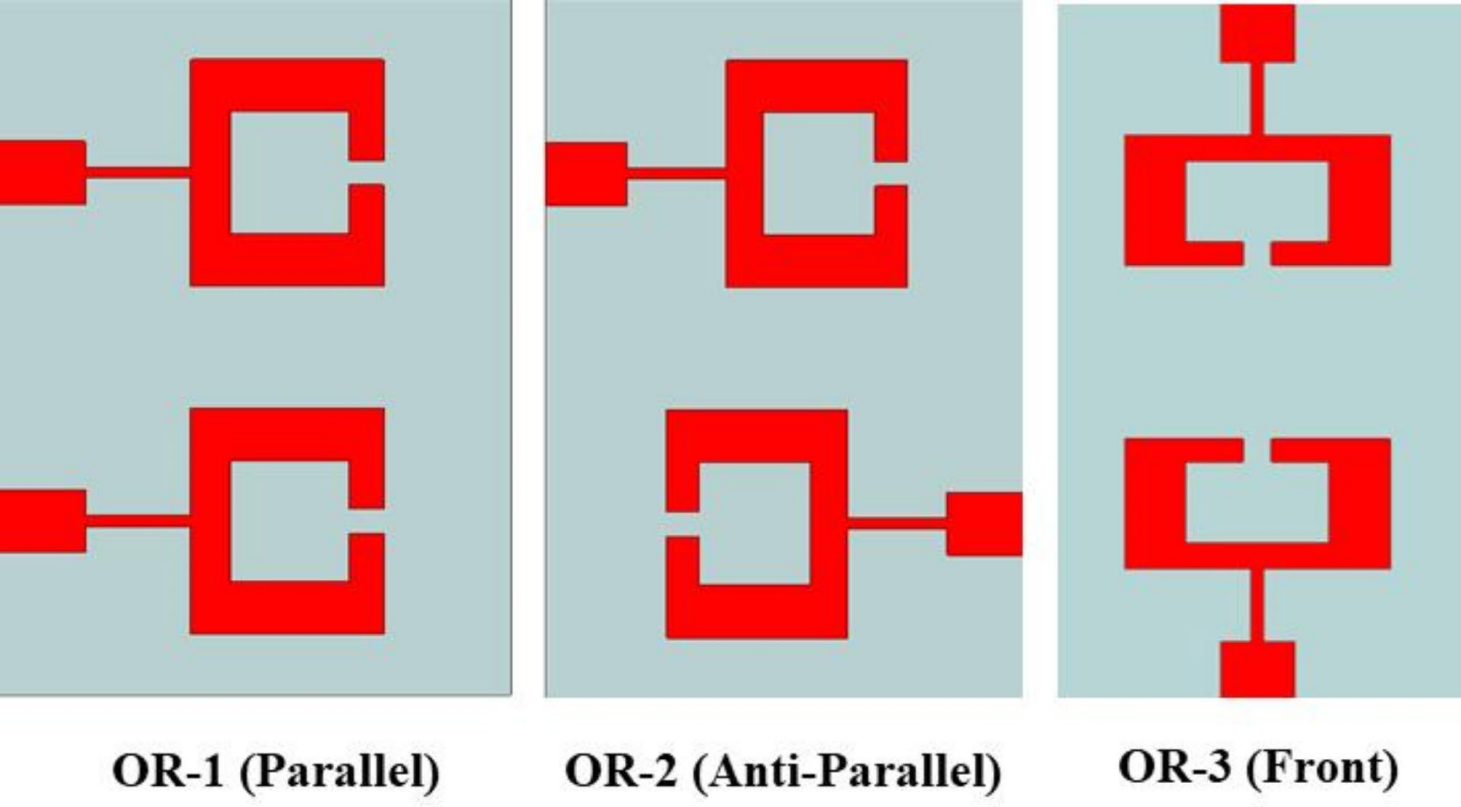
Different Orientation of the MIMO Antenna.

This study suggests that the Taguchi-based Neural Network (Taguchi NN) approach, when applied to the problem of predicting the reflection coefficient based on the independent variables of gap width ∆w, gap shift ∆t, and frequency f (28–34.5 GHz), is effective tool optimize the structure of capacitive loading antenna without the need of simulation and the antenna designers can forecast the outcome at the desired millimeter wave frequency bands.

## MIMO antenna design and e-field distribution

**Fig. 10:**
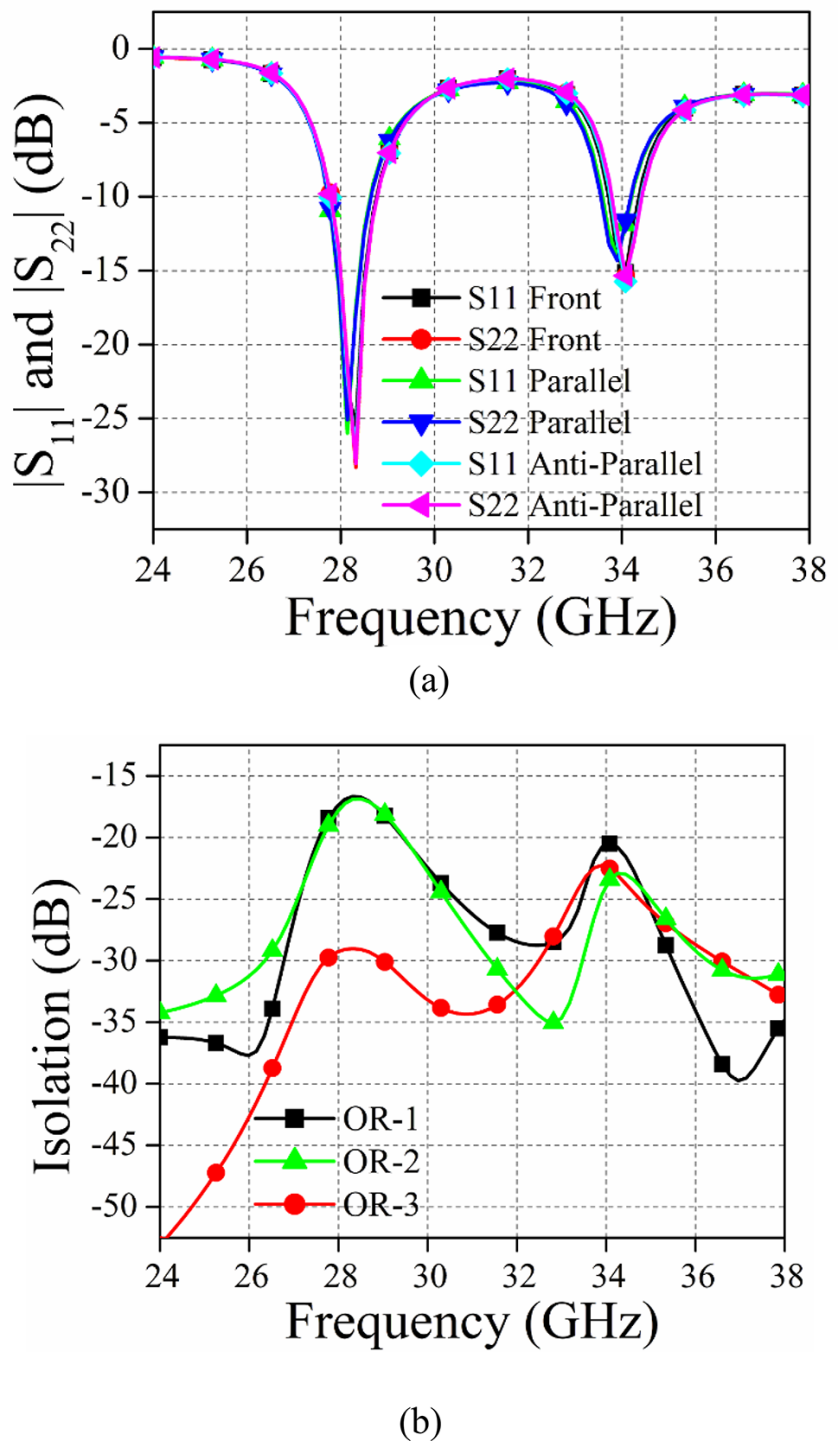
(a) displays the scattering parameters (|S_11_| and |S_22_|) and (b) shows the isolation (|S12|) for various MIMO antenna orientations.

The suggested 2-port MIMO system is achieved by replicating the single antenna component in front-to-front configuration. Figure 10 (a) and (b) provide a comparative analysis of several MIMO antennas by replicating the same antenna element in various orientations and evaluating their return loss and isolation, respectively. Figure 9 examines three distinct MIMO antenna orientations. OR-1 corresponds to the parallel positioning of the antenna elements, OR-2 corresponds to the anti-parallel positioning, OR-3 represents the front-to-front placement of the antenna units. Upon examining Fig. 10 (a), it is evident that the variation in |S_11_| and |S_22_| are similar for all orientations.

**Fig. 11:**
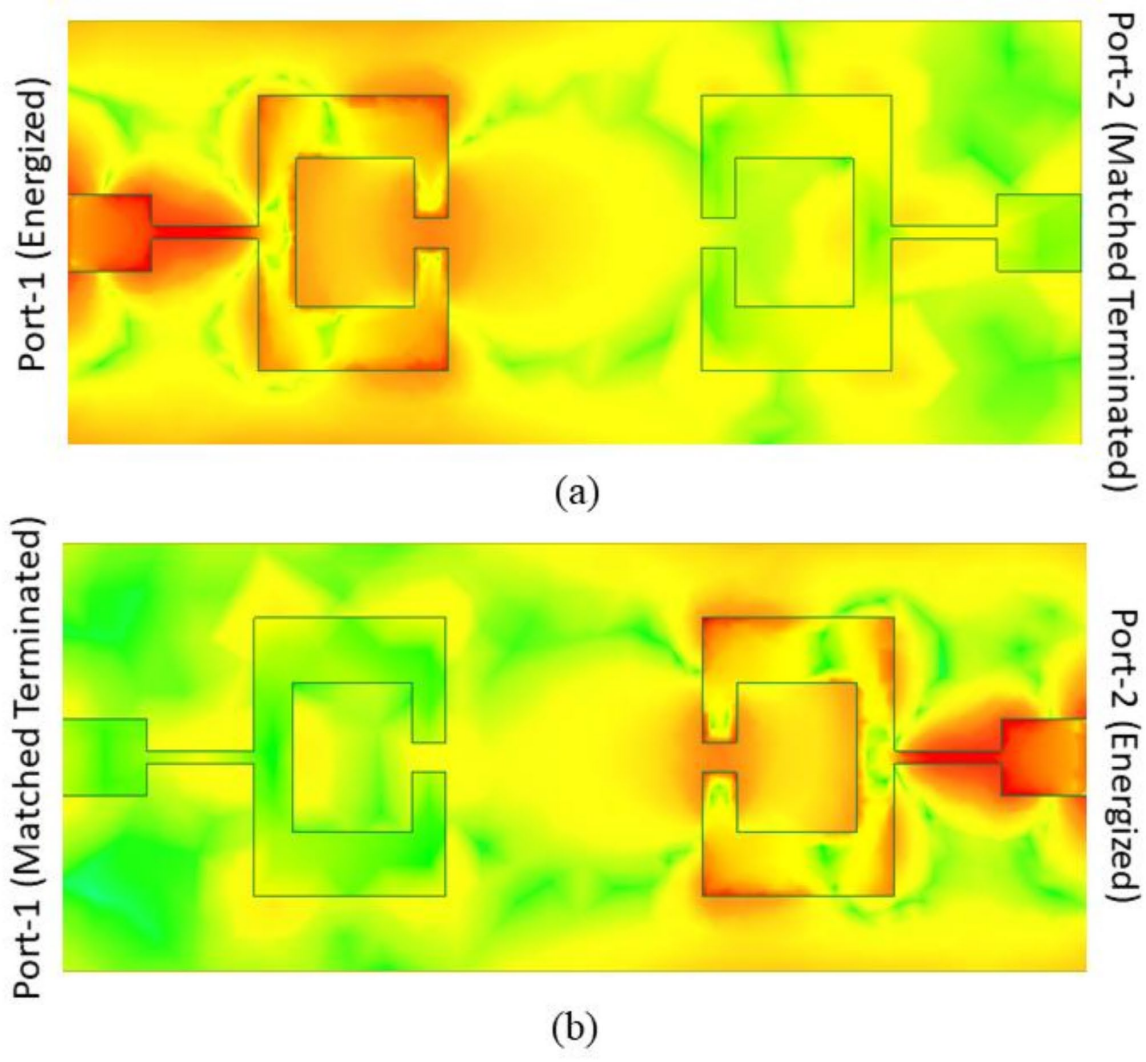
(a) Electric Field Strength with port 1 enabled (b) Electric Field Strength with port 2 enabled.

The electric field intensities at each port of the dual-port MIMO antenna is shown in Fig. 11. Obviously, the area closest to the building’s origin has the strongest current. This proves that, within the given frequency range, these structural components are the ones most responsible for the structure’s resonance. In addition, the electric field strength along the feed line of every antenna element is rather substantial. Not only that, but there are less coupling fields between the antenna components because of the front-to-front configuration. Here we can see the E-field strength with port 1 alone enabled (Fig. 11 (a)) and with port 2 activated (Fig. 11 (b)).

## Results and discussion

In order to verify the properties of the suggested antenna, a prototype was constructed and tested experimentally as seen in Fig. 2. The fabrication method used conventional PCB technology. Ultimately, a thorough examination of both measured and simulated data was carried out, including many performance metrics including reflection coefficient, transmission coefficient, broadside gain, and radiation patterns. This inquiry is further upon in the following sections.

Further, the results of ML study indicate that the Taguchi-based Neural Network (Taguchi NN) approach is highly effective in predicting the reflection coefficient (S_11_) for the proposed antenna across the specified frequency range of 28–34 GHz. The optimal configuration, which involved a neuron setup of (15, 20, 25), a learning rate of 0.001, and a batch size of 20, achieved a validation MSE of 2.244. This low MSE value signifies a close match between the predicted and actual |S_11_| values, indicating that the model has successfully captured the underlying relationship between the input parameters (gap width ∆w, gap shift ∆t, and frequency f) and the reflection coefficient.

### Scattering parameters and gain/radiation efficiency

**Fig. 12:**
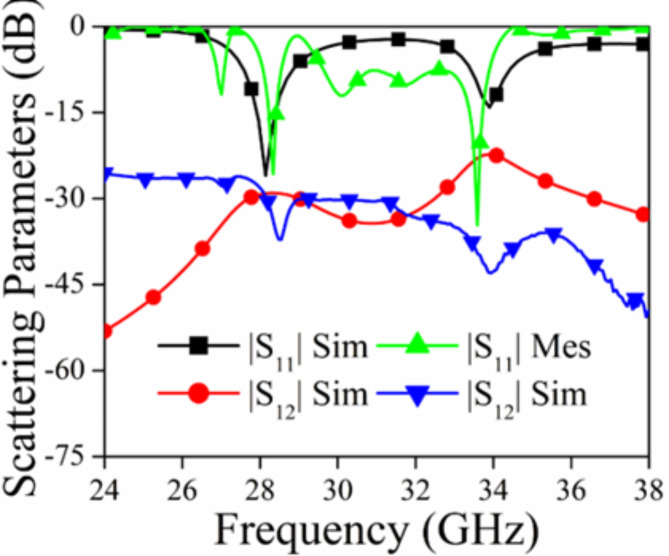
|S_11_|, |S_12_| of the Proposed MIMO Antenna.

**Fig. 13:**
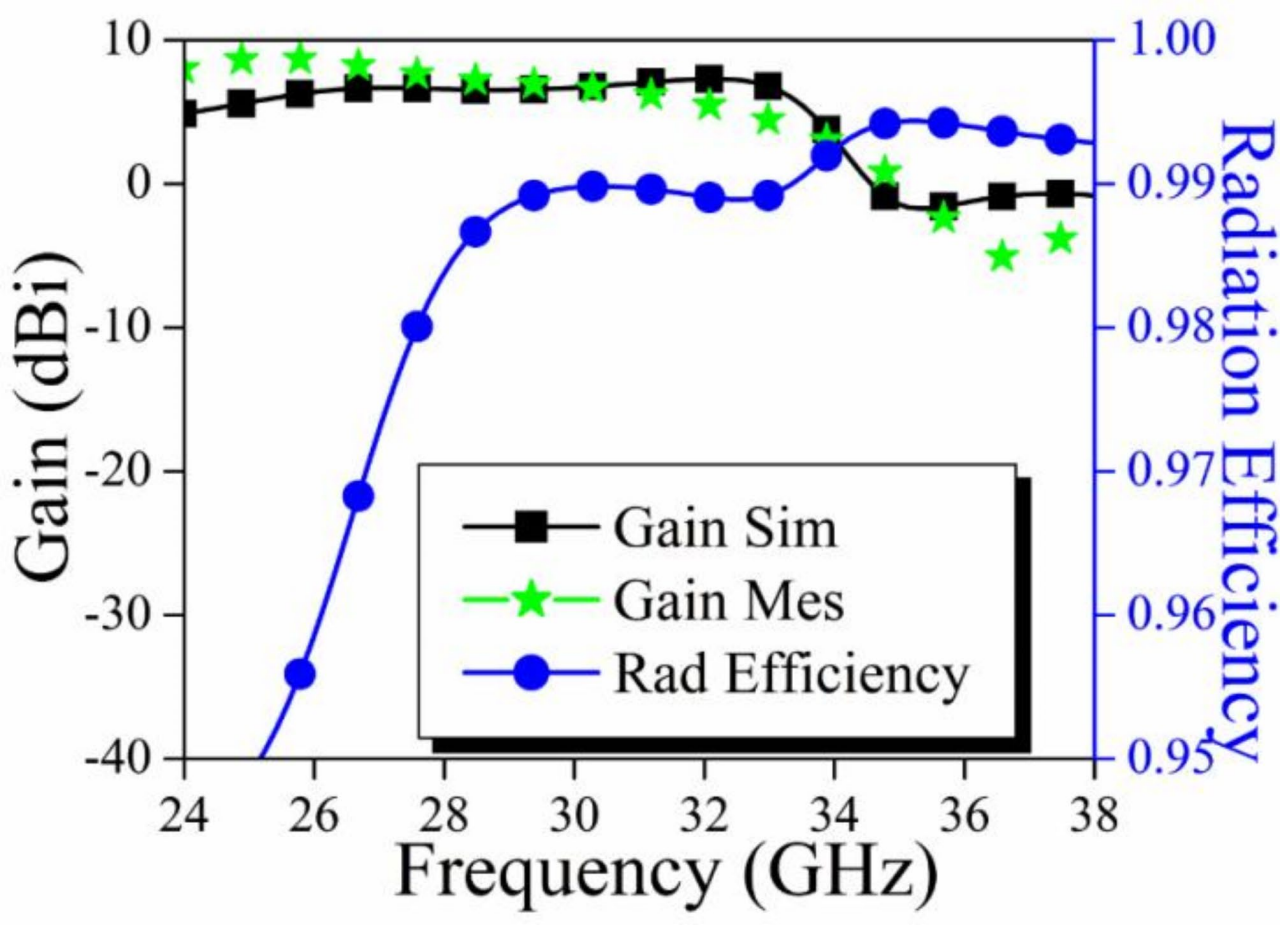
Gain and Radiation efficiency plot of the Proposed MIMO Antenna.

Figure 12 illustrates the predicted and measured outcomes of the recommended antenna. The antenna functioned with the dual frequency band of 28 GHz and 34 GHz, as determined by modeling findings and the simulated isolation levels are observed less than 30 dB and 23 dB, respectively. The antenna had satisfactory performance, as shown by the measured findings, throughout the frequency ranges of 27.61–28.49 GHz and 33.61–34.27 GHz. The measured isolation levels were below 30 dB and 40 dB, respectively. The concordance between the measured and simulated findings is evident. The gain and radiation efficiency plot for the proposed antenna is shown in Figure 13. From the observation of the plot, it is evident that the value of the gain is more than 8.75 dBi for 28 GHz and 5.5 dBi for 34 GHz, while the radiation efficiency is more than 98.5% for both the operating bandwidth.

### Radiation Pattern

**Fig. 14:**
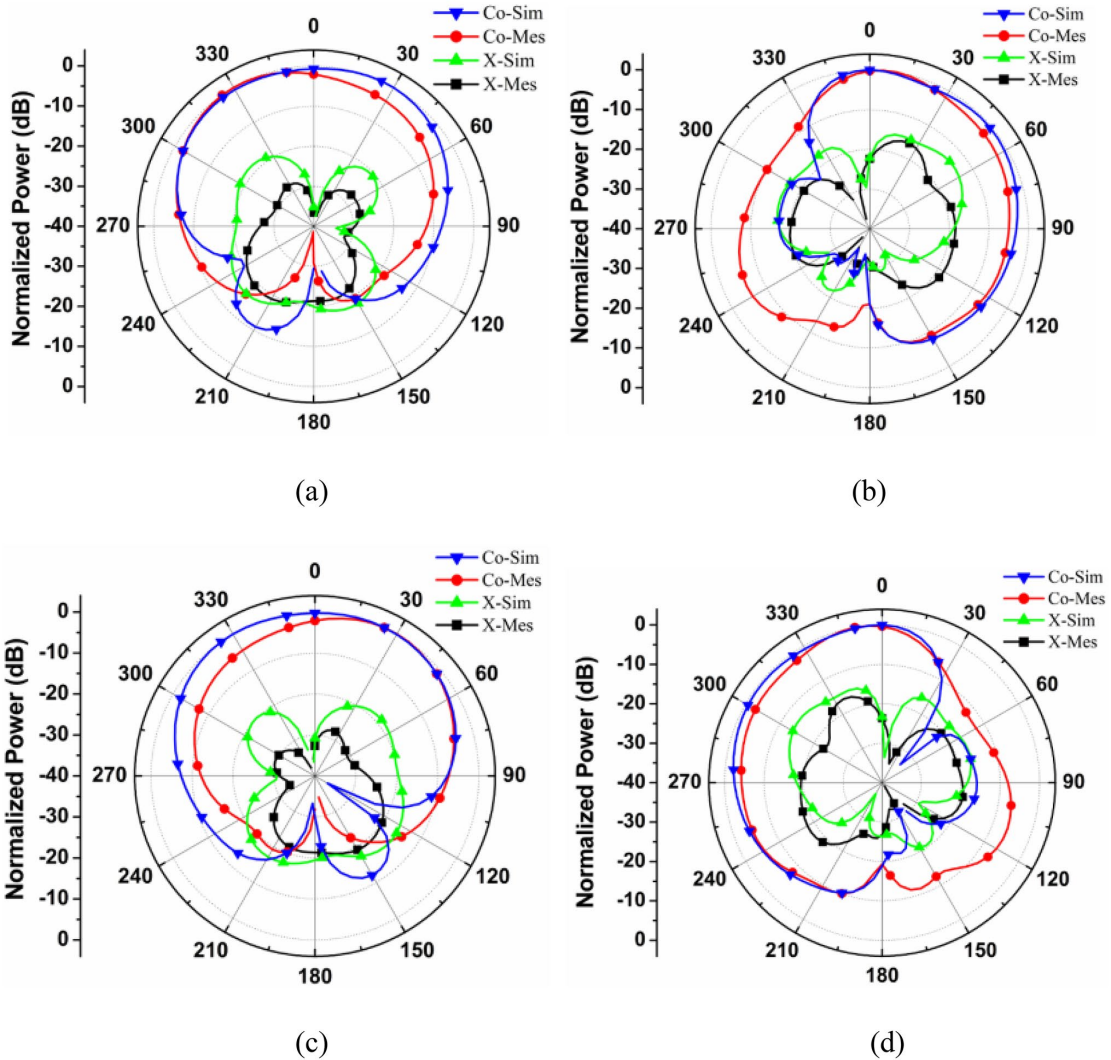
Co and Cross Polarization Plot for the Proposed MIMO antenna at 28 GHz (a) 0° Plane at Port 1, (b) 90° Plane at Port 1, (c) 0° Plane at Port 2, (b) 90° Plane at Port.

**Fig. 15:**
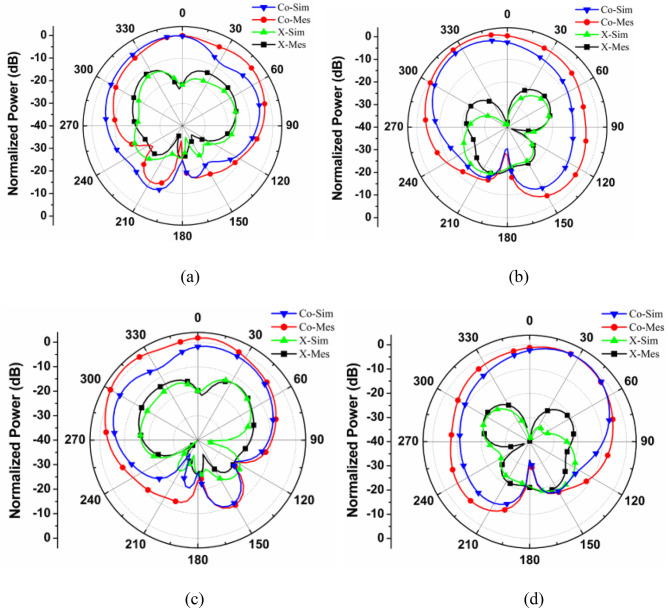
Co and Cross Polarization Plot for the Proposed MIMO antenna at 34 GHz (a) 0° Plane at Port 1, (b) 90° Plane at Port 1, (c) 0° Plane at Port 2, (b) 90° Plane at Port 2.

The high-frequency electromagnetic simulation using the commercial tool HFSS is used to determine the far-field radiation pattern of the proposed MIMO antenna system and the same has been verified with the help of a measured counterpart of the fabricated prototype. The simulated radiation patterns at 28 and 34 GHz are shown in Figs. 14 and 15, respectively, in the major planes *ϕ* = 0◦ and *ϕ* = 90◦, using one port as the excitation source and other port is matched terminated. Sufficient isolation is observed between the Co-polarized and Cross-polarized waves for both frequency bands.

### Diversity parameters

The performance of multi-channel propagation and the autonomy of each port in the proposed MIMO antenna are investigated and understood via the computation of various diversity performance metrics. Total active reflection coefficients (TARCs), diversity gain (DG), and envelope correlation coefficient (ECC) are among the measures that have been considered. The ECC, or element correlation coefficient, is a crucial metric for MIMO (multiple-input multiple-output) systems. It quantifies the degree of independence between antenna elements based on their particular properties. The ECC characteristics of each antenna element may be determined by calculating them using the provided equations based on the simulated findings as mentioned in Eq. 1^26,27^.

1 $$EC{C_s}={\left| {\frac{{\left| {S_{{11}}^{ * }{S_{12}}+S_{{21}}^{ * }{S_{22}}} \right|}}{{{{\left| {\left( {1 - {{\left| {{S_{11}}} \right|}^2} - {{\left| {{S_{21}}} \right|}^2}} \right)\left( {1 - {{\left| {{S_{22}}} \right|}^2} - {{\left| {{S_{12}}} \right|}^2}} \right)} \right|}^{1/2}}}}} \right|^2}$$

**Fig. 16:**
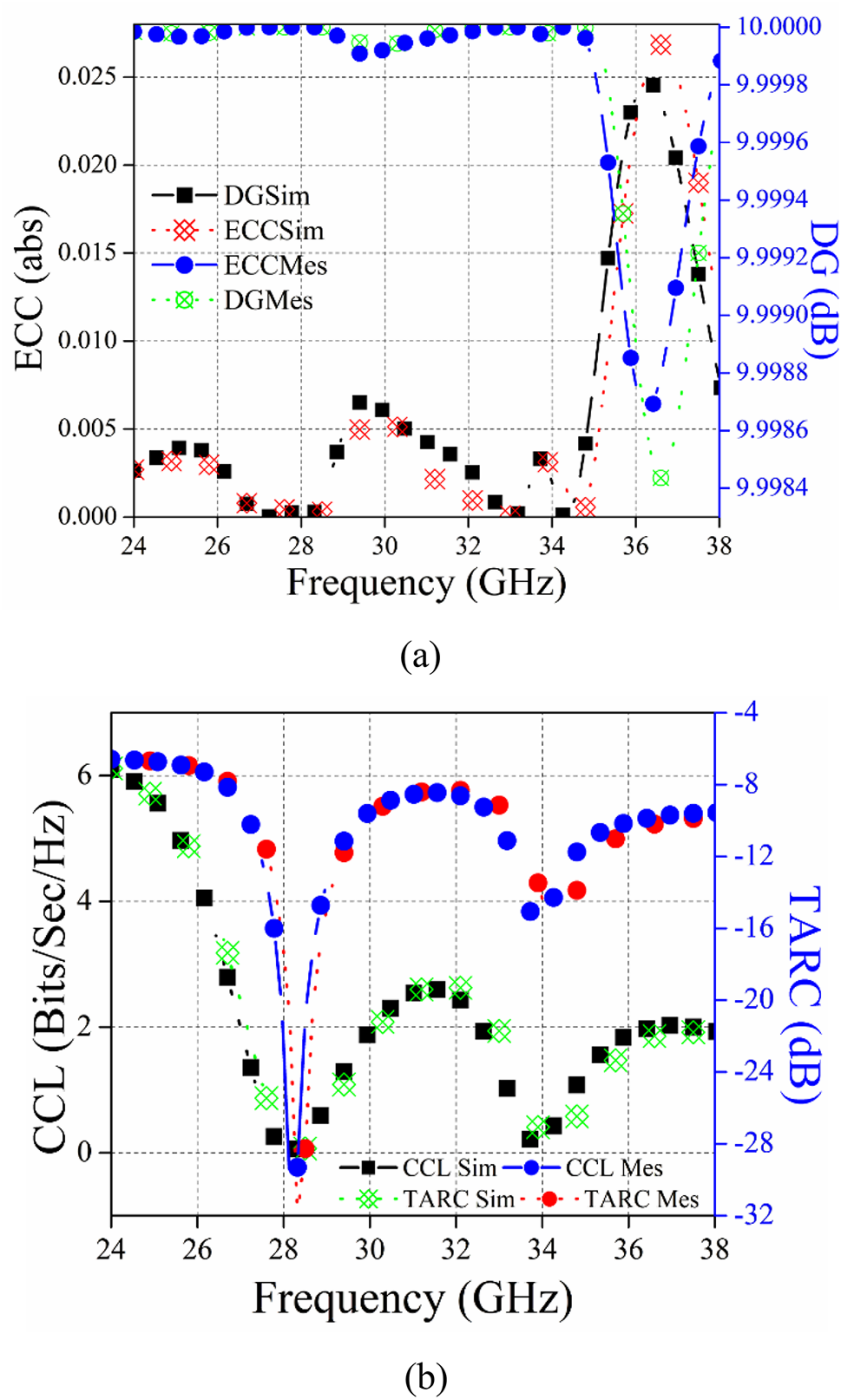
Diversity performance parameters of the MIMO antenna (a) ECC, DG, (b) CCL, TARC.

Figure 16 (a) depicts the simulated results for the proposed two-port dual-band multiple-input multiple-output (MIMO) antenna using ECC. The ECC value is less than 0.0001 in both the first and second bands. The way in which a MIMO system’s antennas are arranged greatly influences the signal-to-interference ratio based on the diversity scheme that is being used. The diversity gain (DG) is calculated to quantify this. It is a crucial component in assessing the performance of the MIMO system. A greater DG value indicates that the multipath propagation’s negative effects are successfully mitigated by the MIMO system, improving signal quality, data throughput, coverage range, and overall system capacity. As a result, the performance may be decreased while maintaining the transmitted power. It is coupled with ECC as Eq. 2^28^.

2 $$DG=10\sqrt {1 - EC{C^2}}$$

The proposed MIMO’s DG is > 9.99 dB for simulated values, as shown in Fig. 16 (a), which is in close proximity to the allowed limit.

Channel capacity loss (CCL) is a vital measure for evaluating the performance of MIMO systems in a multipath environment. The maximum achievable data rate that may be sent successfully across a communication medium is determined. It is preferable for the CCL to be less than 0.3 bit/s/Hz. The calculation of CCL may be derived using Eq. 3.

3 $$\begin{aligned} {C_{loss}} &= - {\log _2}\det ({{\uppsi}^R}) \\ & {{{{\uppsi}}}^R}=\left( {\begin{array}{*{20}{c}} {{\psi _{11}}}&{{\psi _{12}}} \\ {{\psi _{21}}}&{{\psi _{22}}} \end{array}} \right) ,{\psi _{11}}=1 - \left( {{{\left| {{S_{11}}} \right|}^2}+{{\left| {{S_{12}}} \right|}^2}} \right) \\& {\psi _{22}}=1 - \left( {{{\left| {{S_{22}}} \right|}^2}+{{\left| {{S_{21}}} \right|}^2}} \right),{\psi _{12}}= - \left( {S_{{11}}^{*}{S_{12}}+S_{{21}}^{*}{S_{12}}} \right) \hfill \\& {\psi _{21}}= - \left( {S_{{22}}^{*}{S_{21}}+S_{{12}}^{*}{S_{21}}} \right) \end{aligned}$$

In Fig. 16 (b), the CCL is computed and shown versus frequency. It is shown that in the 28-GHz and 34-GHz bands, the suggested MIMO system has the best CCL (lowest) values. A CCL value of 0.005 is noted. When determining the optimal approach to multiport antenna design, TARC is still another crucial consideration. It illustrates the relative amounts of electricity that are incident and reflected. For a MIMO antenna with two ports, the value of this parameter may be calculated using Eq. 4^29^.

4 $$\Gamma _{a}^{t}={\raise0.7ex\hbox{${\sqrt {\sum {_{i}^{N}{{\left| {{b_i}} \right|}^2}} } }$} \!\mathord{\left/ {\vphantom {{\sqrt {\sum {_{i}^{N}{{\left| {{b_i}} \right|}^2}} } } {\sqrt {\sum {_{i}^{N}{{\left| {{a_i}} \right|}^2}} } }}}\right.\kern-0pt}\!\lower0.7ex\hbox{${\sqrt {\sum {_{i}^{N}{{\left| {{a_i}} \right|}^2}} } }$}}$$

Figure 16 (b) displays the calculated Total Active Reflection Coefficient (TARC) of the antenna design being presented.

Table 3, presents a comparison between the proposed work and other recent publications that are relevant in the literature. The MIMO antenna designs described in^1,30–33^ are generally bigger than the intended antenna, making them unsuitable for use with modern compact communication devices. Nevertheless, only a limited number of devices have successfully attained a compact configuration with low gain. In addition, the isolation achieved by these MIMO designs is inadequate when compared to the recommended MIMO antenna. It is worth mentioning that, unlike this research, earlier studies only focused on evaluating the impact of ECC and DG on MIMO performance. Consequently, the antenna design proposed in this study surpasses current designs and demonstrates its compatibility with current and future communication systems. The device has a compact shape, significant amplification, MIMO functionalities, and commendable diversity efficiency.

**Table 3:** Research comparing the suggested 2 Port MIMO antenna for millimeter wave applications to the previously documented dual Band MIMO antenna.

Ref.	Frequency (GHz)	Dimension (mm × mm)	Gain (dBi)	Isolation (dB)	ECC (abs)	DG (dB)
^30^	28/38	55 × 110	7/8	27	NA	NA
^31^	28/38	30 × 30	5.2	30	0.0001	9.99
^32^	28/38	55 × 110	7.8/8.2	28	0.002	NA
^1^	28/38	27.65 × 12	5.2/5.3	30/22	0.0001	9.99
^33^	27/39	26 × 11	5/5.7	30/25	0.0001	NA
**Prop.**	**28/34**	**24 × 10**	**8.75/5.5**	**30/25**	**0.0001**	**9.99**

## Conclusion

This paper presents a compact microstrip patch antenna designed specifically for 5G mobile phones, operating at dual frequencies of 28/34 GHz. The study concludes that the Taguchi-based Neural Network (Taguchi NN) is a highly effective tool for optimizing the design of mm wave antennas in the 28–34.5 GHz frequency range. The model demonstrated strong predictive accuracy with low MSE and high R² scores, enabling reliable prediction of the reflection coefficient (S11) without extensive simulations. This approach offers a streamlined and efficient method for antenna designers to achieve optimal performance in millimeter-wave applications. The designed antenna has been optimized to operate within the frequency ranges of 27.61–28.49 GHz and 33.61–34.27 GHz. It achieves an isolation level of more than 30 dB for 28 GHz and 25 dB for 34 GHz. In order to validate the antenna design and showcase the exceptional quality of the suggested two-element MIMO antenna, MIMO parameters such as ECC, DG, and CCL have been derived using simulation data. Also, XG boost can be implemented to predict the reflection coefficient which provides a satisfactory prediction outcome. The results obtained from the simulation and testing indicate a favorable pattern within the two operating frequency ranges, suggesting that the proposed architecture is suitable for 5G communications.

## Data Availability

The datasets used and/or analyzed during the current study available from the corresponding author on reasonable request.
